# Genetic diagnosis of endocrine disorders in Cyprus through the Cyprus Institute of Neurology and Genetics: an ENDO-ERN Reference Center

**DOI:** 10.1186/s13023-024-03171-4

**Published:** 2024-04-18

**Authors:** Vassos Neocleous, Pavlos Fanis, Meropi Toumba, Nicos Skordis, Leonidas A. Phylactou

**Affiliations:** 1 Department of Molecular Genetics, Function and Therapy, The Cyprus Institute of Neurology and Genetics, Nicosia, Cyprus; 2 Department of Pediatrics, Pediatric Endocrinology Clinic, Aretaeio Hospital, Nicosia, Cyprus; 3 Division of Paediatric Endocrinology, Paedi Center for Specialized Paediatrics, Nicosia, Cyprus; 4 School of Medicine, University of Nicosia, Nicosia, Cyprus

**Keywords:** ENDO-ERN, Endocrine disorders, CAH, MEN2, *RET*, *MKRN3*, DSD, MODY

## Abstract

The report covers the current and past activities of the department *Molecular Genetics-Function and Therapy (MGFT)* at the *Cyprus Institute of Neurology and Genetics (CING)*, an affiliated Reference Center for the European Reference Network on Rare Endocrine Conditions (Endo-ERN).

The presented data is the outcome of > 15 years long standing collaboration between *MGFT* and endocrine specialists from the local government hospitals and the private sector. Up-to-date > 2000 genetic tests have been performed for the diagnosis of inherited rare endocrine disorders. The major clinical entities included Congenital Adrenal Hyperplasia (CAH) due to pathogenic variants in *CYP21A2* gene and Multiple Endocrine Neoplasia (MEN) type 2 due to pathogenic variants in the *RET proto-oncogene*. Other rare and novel pathogenic variants in *ANOS1, WDR11, FGFR1, RNF216*, and *CHD7* genes were also found in patients with Congenital Hypogonadotropic Hypogonadism. Interestingly, a few patients with Disorders of Sexual Differentiation (DSD) shared rare pathogenic variants in the *SRD5A2*, *HSD17B3* and *HSD3B2* while patients with Glucose and Insulin Homeostasis carried theirs in *GCK* and *HNF1A* genes. Lastly, *MGFT* over the last few years has established an esteemed diagnostic and research program on premature puberty with emphasis on the implication of *MKRN3* gene on the onset of the disease and the identification of other prognosis biomarkers.

As an Endo-ERN member *MGFT* department belongs to this large European network and holds the same humanistic ideals which aim toward the improvements of health care for patients with rare endocrine conditions in respect to improved and faster diagnosis.

## Introduction

The recent scientific developments in biotechnology and genetics have had a distinctive impact on the diagnosis of inherited and long-term diseases including the inherited endocrine disorders [1]. The broadly used over the last couple of decades ‘gold standard’ Sanger sequencing and the most recent and emerging high throughput next-generation sequencing (NGS) approaches are the main reasons that have led to the identification of genetic variants with an unparalleled impact on the management of the affected patient and their relatives. Since January 2020, the accredited with ISO 15189 department of *MGFT* at the *CING* has become an affiliated Reference Center for the Endo-ERN (https://endo-ern.eu/reference-centre/department-of-molecular-genetics-function-and-therapy-the-cyprus-institute-of-neurology-and-genetics/). Currently, *MGFT* is participating in three of the Main Thematic Groups (MTG1_Adrenal; MTG6_Hypothalamic and Pituitary conditions; MTG7_Sex Development & Maturation) of Endo-ERN network and contributes to the main objective and shared goal of the network which is to improve access to high-quality healthcare for patients with rare hormonal disorders. For this purpose, *MGFT* along with the other Reference Centers across Europe actively participates to the continuous education programs that are regularly offered virtually or organized in the format of scientific meetings by the Network. Additionally, *MGFT* is in direct contact with a great number of the other Reference Centers and regularly shares and receives valuable clinical and scientific information so as to ensure that the finest care is made available to the patients both locally and across Europe. Lastly, *MGFT* has established distinctive and ongoing basic science research programs on the molecular mechanisms of pubertal development (unpublished data) and epidemiological surveillances on specific rare endocrine disorders such as congenital adrenal hyperplasia [2–5].

The experience of *MGFT* on the diagnosis and research of inherited endocrine disorders is long standing and dates back to the mid-2000s, and since then has been effectively using at its state-to-the-art facilities the most recent technologies necessary for the comprehensive diagnosis of patients with a series of inherited endocrinopathies. Such disorders include CAH [3], DSD [6–10], MEN type 2A and 2B [11], genetic conditions causing hypogonadotropic hypogonadism [12, 13], precocious and delayed puberty [4, 14–17], maturity onset diabetes of the young (MODY) [18], cases of obesity and several other less frequent disorders [19, 20]. The diagnosis of inherited rare conditions is always challenging and the current diagnostic genetic approaches have become indispensable tools for the daily practice of pediatric and adult endocrinology. Consequently, to a great extent the evaluation of the phenotype and the management of the disease could rely on them. Over the last few years the overall diagnostic outcome of our department with the implementation of NGS assays has significantly increased to the benefit of the Cypriot patients with complicated inherited endocrinopathies.

### Diagnostic endocrine services offered by *MGFT* at *CING—ENDO-ERN* affiliated Reference Center

#### *Molecular diagnosis of CAH* due to *CYP21A2* pathogenic variants

**Fig. 1:**
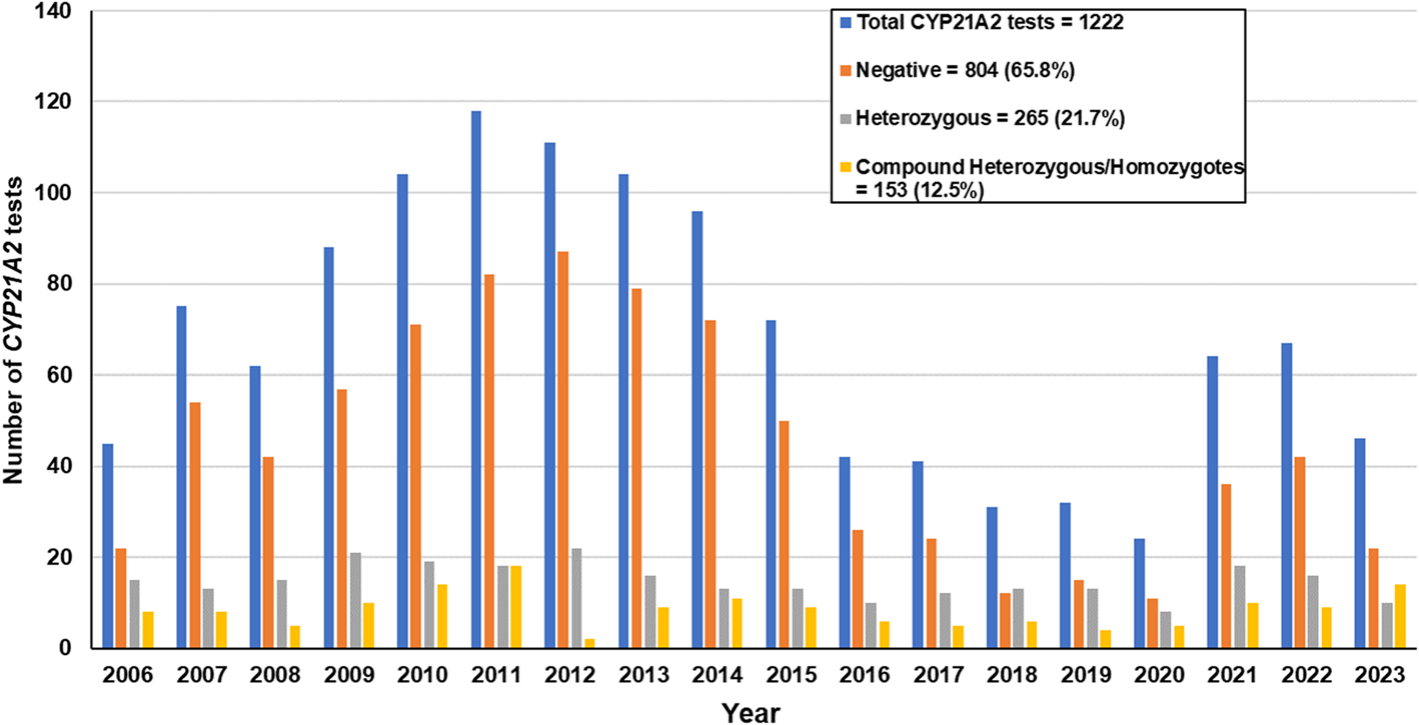
Graph illustrating the number of patients referred to *MGFT* for genetic diagnosis of *CYP21A2* from 2006 to July 2023. A total of 1222 patients with clinical suspicion of CAH have been tested since 2006, two-hundred and sixty-five (21.7%) were identified as heterozygous and 153 (12.5%) as compound heterozygous/homozygous

**Fig. 2:**
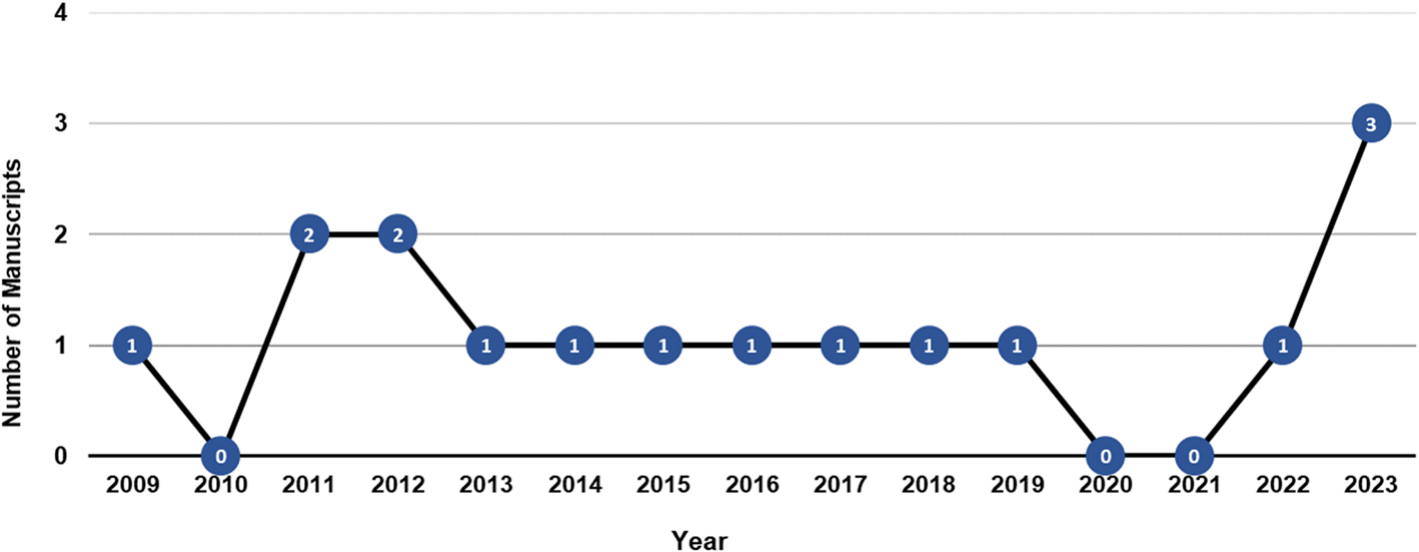
Since the year 2009, a total number of sixteen manuscripts that covered extensive clinical, genetic, epidemiological and other scientific information of CAH in Cyprus have been published by the *MGFT* department of the Cyprus Institute of Neurology and Genetics

In January 2006, the *MGFT* department at *CING* introduced in the Republic of Cyprus the carrier screening of the *CYP21A2* gene for the diagnosis of 21-hydroxylase deficiency (21-OHD), the most common cause of CAH [21]. The clinical phenotypes of CAH as a result of mutations in the *CYP21A2* gene ranges from the classic most severe salt-wasting (SW) and simple virilizing (SV) forms with prenatal virilization in females, to the milder and more frequent non-classic form [22]. Both classic SW and SV forms of the disorder are rare and characterized by prenatal virilization in females, their estimated frequency in most populations range between 1:10,000 to 1:20,000 [22]. Nowadays, the estimate for the non-classic and milder form of the disorder which is characterized by no glucocorticoid deficiency ranges between 1:100–1:500 live births [3, 22]. In general, female individuals with the non-classic form carry in compound heterozygosity two mild *CYP21A2* pathogenic variants and present symptoms of hyperandrogenemia, hirsutism, premature puberty in early life and or infertility later on in life [23]. Several studies including one from our group have estimated the carrier incidence of the *CYP21A2* variants in the general population to be 1:10–1:25 [2]. Heterozygous *CYP21A2* gene carriers can also be identified with the ACTH-stimulated 17-OH Progesterone (17-OHP) values, although in general for this specific test there is a greater overlap with the healthy population. Recent studies including a couple by our department have demonstrated that a significant percentage of female *CYP21A2* heterozygous carriers can be at increased risk of developing hyperandrogenemia and that indeed there are discrepancies in hormonal levels among heterozygous carriers, non-carriers, and females with non-classic congenital hyperplasia [24–27]. Since 2006, the accredited with ISO 15189 MGFT at the *CING* has performed more than 1200 diagnostic *CYP21A2* genotypic analyses (Fig. 1). The genetic analyses were referred to *MGFT* by the national reference hospitals of the republic of Cyprus and to a lesser extent from the private sector. The great majority of these patients that underwent genetic testing for *CYP21A2* pathogenic variants were females with hyperandrogenism either in prepubertal or in peripubertal ages (premature pubic hair development, bone age advancement, severe acne and/or hirsutism, with or without menstrual irregularity, and complete lack of virilization with increased 17-OHP levels). This collaboration resulted into an extensive publication record that mostly covered clinical and genetic findings [3, 25, 26, 28–36] (Fig. 2), epidemiological data regarding the frequency of *CYP21A2* in the local population [2] and other scientific information regarding the multiallelic and tandem RCCX complex in the *MHC class III* region of chromosome 6p21.3 [37, 38] (Fig. 2). As per the recent CAH Best Practice Guidelines [39], the *MGFT* department is currently using for the genetic investigation of the *CYP21A2* gene the gold standard method of Sanger sequencing and the multiplex ligation-dependent probe amplification (MLPA) [2, 3, 26, 37, 38]. In addition to MPLA that is the most appropriate method for the detection of rare duplications/deletions, we have extended the *CYP21A2* genetic investigation by employing the *TaqI* digestion, which also detects the presence of the chimeric *CYP21A1P/CYP21A2* and *TNXA/TNXB* gene deficiencies and overcomes a number of limitations of the Sanger sequencing and MLPA methods.

**Fig. 3:**
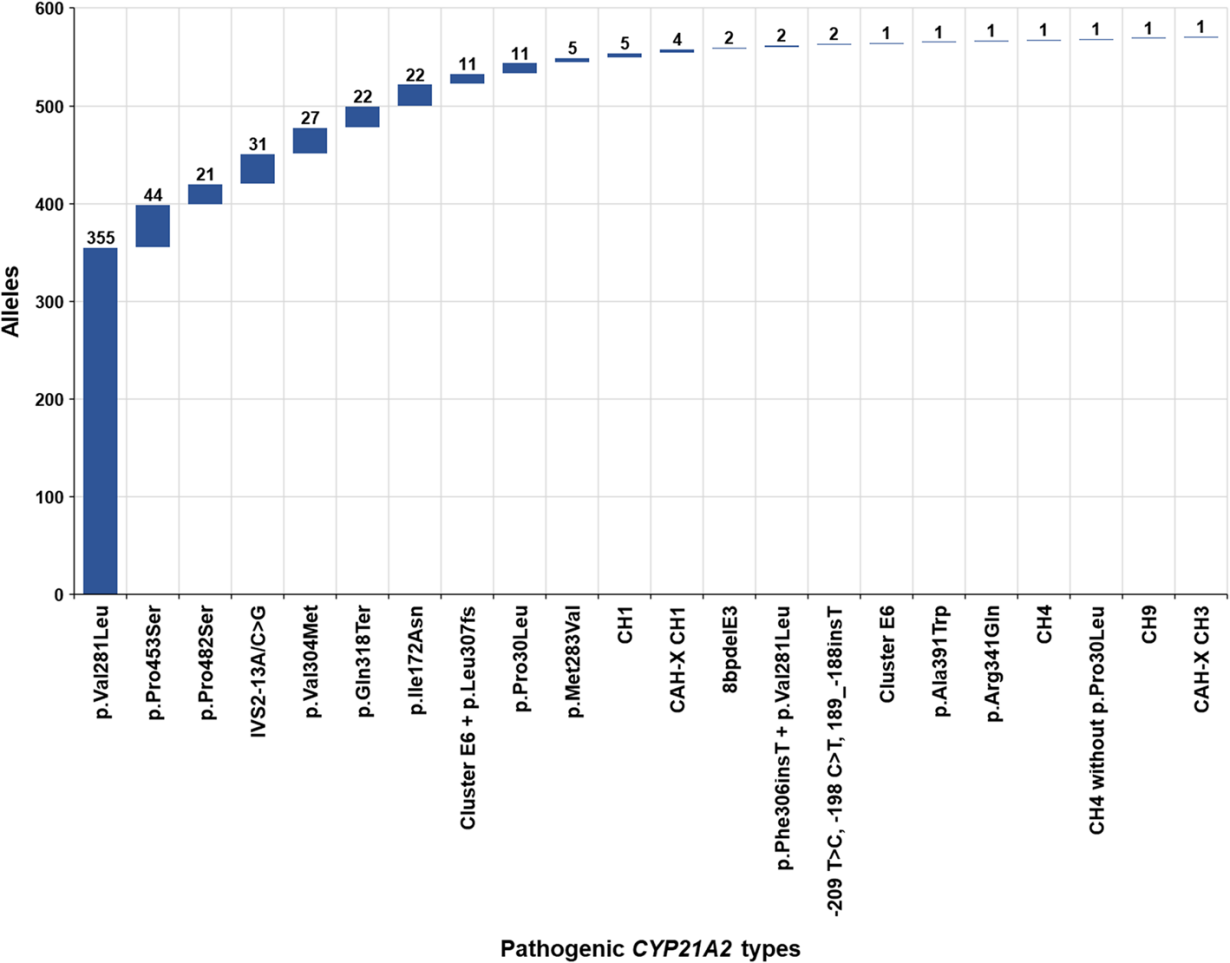
Waterfall plot of the all identified *CYP21A2* pathogenic variants in Cyprus. A total of 571 pathogenic variants have been identified, with p.Val281Leu to be the most frequent (62.17%)

Up-to-date a total number of 571 pathogenic *CYP21A2* variants have been identified, with the non-classic p.Val281Leu to be the most prevalent (62.17%) (Fig. 3). A comprehensive analysis of the *CYP21A2* allelic prevalence in the cohort of patients that included heterozygotes (*n* = 265), compound heterozygotes/homozygotes (*n* = 153) is depicted in the waterfall plot in Fig. 3. It should be noted that up-to-date in Cyprus a total number of 18 patients with the severe classic form of the disease were born, a detailed genotype/phenotype correlation analysis of the CAH patients with the SW and the SV form is depicted in Table 1. There are multiple recent and earlier studies that describe the exerted pathogenic effect(s) of the identified *CYP21A2* gene variants and up-to-this time are in alliance and similar with the observed clinical phenotypes of our tested patients at *MGFT* department. Subsequently on this issue, we are in direct contact with the ENDO-ERN network and we regularly share our findings with other reference centers in webinars, meetings and electronic databases such as the European Registries for Rare Endocrine Conditions (EuRRECa) (https://endo-ern.eu/registries/eurreca/). Moreover, *MGFT* department participated in a recent study/survey on the current reflection of the expert views across Europe on the importance of the prenatal dexamethasone (Pdex) treatment of CAH due to 21-OH deficiency [35]. Information regarding the epidemiological frequencies of the identified *CYP21A2* pathogenic variants and their associated pathogenic effects can be obtained from the *Human Cytochrome P450 Allele Nomenclature database* (https://www.pharmvar.org/htdocs/archive/cyp21.htm), the *Human Gene Mutation Database* (https://www.hgmd.cf.ac.uk/ac/all.php) and by the recent by European Molecular Genetics Quality Network (EMQN) best practice guidelines for molecular genetic testing and reporting of 21-OHD [39]. This study/review did not involve patients; therefore, no patient consent was required. The study though was approved by the Cyprus National Ethics Committee (EEBK/ΕΠ/2016/28).

**Table 1:** A detailed genotype/phenotype correlation analysis regarding the patients with the classic SW and the SV form of CAH. All patients underwent genetic testing at the accredited with ISO 15189 *MGFT* department of the *CING*

	Pathogenic Variants in the *CYP21A2* gene	21-Hydroxylase Form	Sex	Age at diagnosis	Clinical Diagnosis	Basal 17-OHP nmol/lt	Hyponatremia -Hyperkalemia
1	p.Pro30Leu/p.Pro30Leu	Simple Virizing	F	6.5 yrs	Clitoromegaly – Premature pubarche	> 25 ng/ml	Negative
2	IVS2-13A/C > G/IVS2-13A/C > G	Salt Wasting	F	Neonate	Prader 3	> 25 ng/ml	Positive
3	IVS2-13A/C > G/IVS2-13A/C > G	Salt Wasting	F	Neonate	Prader 3	> 25 ng/ml	Positive
4	IVS2-13A/C > G/IVS2-13A/C > G	Salt Wasting	M	Neonate	Adrenal crisis	> 25 ng/ml	Positive
5	IVS2-13A/C > G/IVS2-13A/C > G	Salt Wasting	M	Neonate	Adrenal crisis	> 25 ng/ml	Positive
6	IVS2-13A/C > G/IVS2-13A/C > G	Simple Virizing	M	5.5 yrs	GnRH independent—Precocious puberty	> 25 ng/ml	Negative
7	CH1/CH4	Simple Virizing	M	6.5 yrs	GnRH independent—Precocious puberty	> 25 ng/ml	Negative
8	IVS2-13A/C > G/CH1	Salt Wasting	M	Neonate	Adrenal crisis	> 25 ng/ml	Positive
9	IVS2-13A/C > G/CAH-X CH1	Salt Wasting	F	Neonate	Ambiguous genitalia -Prader 5	> 25 ng/ml	Positive
10	IVS2-13A/C > G/p.Gln318stop	Salt Wasting	F	Neonate	Ambiguous genitalia	> 25 ng/ml	Positive
11	p.Phe306insT + p.Val281Leu/p.Phe306insT + p.Val281Leu	Salt Wasting	F	Neonate	Prader 4	> 25 ng/ml	Positive
12	CAH-X CH1/CAH-X CH1	Salt Wasting	M	Neonate	Adrenal crisis	> 25 ng/ml	Positive
13	CH1/CH1	Salt Wasting	M	Neonate	Adrenal crisis	> 25 ng/ml	Positive
14	CH1/p.Gln318stop	Salt Wasting	M	Neonate	Adrenal crisis	> 25 ng/ml	Positive
15	p.Ile172Asn/CAH-X CH3	Simple Virizing (SV)	M	Neonate	Ambiguous genitalia	> 25 ng/ml	Negative
16	p.Ile172Asn/p.Ile172Asn	Simple Virizing	F	4.5 yrs	Ambiguous genitalia	> 25 ng/ml	Negative
17	p.Ile172Asn/p.Ile172Asn	Simple Virizing	M	5.0 yrs	Ambiguous genitalia	> 25 ng/ml	Negative
18	p.Ile172Asn/p.Ile172Asn	Simple Virizing	M	3.2 yrs	Ambiguous genitalia	> 25 ng/ml	Negative

#### Molecular diagnosis of multiple endocrine neoplasia type 2 (MEN 2) due to RET proto-oncogene pathogenic variants

**Fig. 4:**
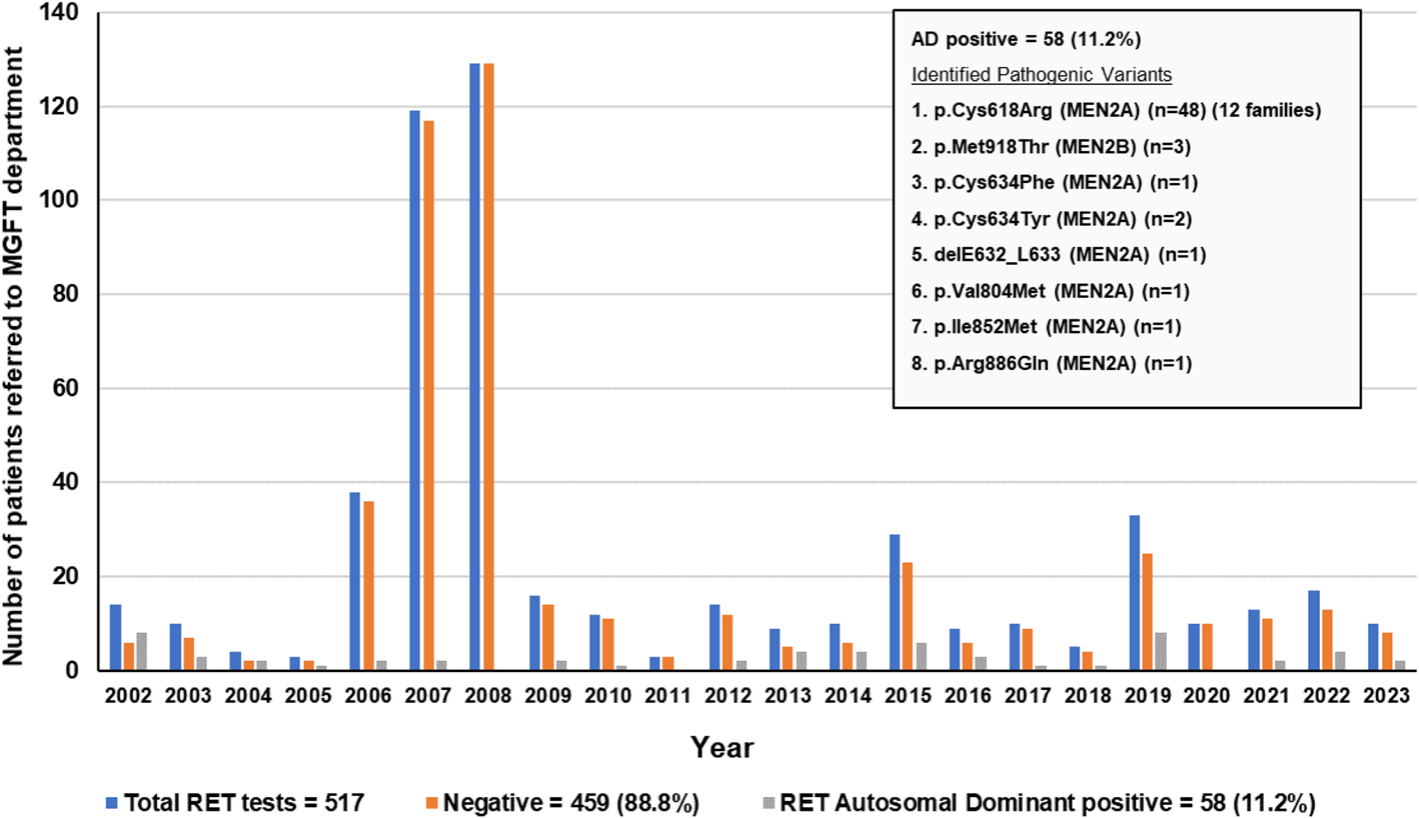
Graph illustrating the number of patients referred to *MGFT* for *RET proto-oncogene* genetic diagnosis. A total of 517 patients with suspected as having MEN2 have been tested since 2002 and 58 (11.2%) were identified to share an AD pathogenic variant in the *RET proto-oncogene*. All identified pathogenic variants are indicated in the square box within the graph. Haplotype analysis with microsatellite markers demonstrated that all 12 probands and family members with p.Cys618Arg carried a core haplotype due to a possible founder effect in the island of Cyprus

Multiple endocrine neoplasia type 2 (MEN2) syndrome is an autosomal dominant (AD) hereditary disorder that is mainly associated with medullary thyroid cancer, parathyroid tumors, and pheochromocytoma [40, 41]. Patients clinically characterized with MEN2 can be distinguished into one of the three distinct forms known as MEN2A, MEN2B and Familial medullary thyroid carcinoma (FMTC). These forms solely rely on the severity of the *RET* *proto-oncogene* pathogenic variants that are inherited [40, 42]. The so far reported MEN2 causing pathogenic variants are classified by the American Thyroid Association (ATA) and the European Thyroid Association (ETA) into the highest (ATA-HST), high (ATA-H), and moderate risk (ATA-MOD) levels and the great majority of them is found in exons 10, 11, 13, 14, 15 and 16 of the *RET* gene [41, 43, 44]. The *MGFT* department is also accredited with ISO 15189 for the genetic test of *RET* proto-oncogene and since 2002 has performed more than 500 tests in patients with clinical MEN2 manifestations i.e. medullary thyroid carcinoma (MTC), pheochromocytoma, hyperparathyroidism and cutaneous lichen amyloidosis (Fig. 4). Up-to-date a total of 58/517 (11.2%) patients from twenty unrelated Cypriot families and two sporadic cases of Russian descent were identified to carry *RET* pathogenic variants and the majority of these findings were perennially presented in a series of manuscripts by our group [11, 45–47]. It is noteworthy to say that among these twenty two unrelated families, twelve probands (54.5%) were heterozygous for the p.Cys618Arg, two for the p.Cys634Tyr (9.1%), one for the somatic delE632_L633 (4.76%), one for the p.Val804Met (4.76%), one for the p.Ile852Met (4.76%), one for p.Arg886Gln (4.5%) and three (13.7%) for the MEN2B p.Met918Thr pathogenic variant (Fig. 4). Back In 2018, we published a manuscript where we provided evidence for a founder effect phenomenon regarding the most frequent p.Cys618Arg pathogenic variant that we identified in a total of twelve nonrelated families of Cypriot descent [11]. More specifically, using a haplotype analysis approach with microsatellite markers we demonstrated that all nine probands and family members with p.Cys618Arg carried a core haplotype [11]. Therefore, for the first time the p.Cys618Arg mutation is suggested to be the result of an ancestral mutation that has spread to the island of Cyprus due to a possible founder effect (Fig. 4). The aggressiveness of MTC in patients with MEN2 correlates with the pheochromocytoma penetrance and the severity of the autosomal dominant (AD) pathogenic *RET proto-oncogene* variants [41]. Prevention therapy of MTC is successfully attained with thyroidectomy once the diagnosis is made or prior the age of probable malignant progression [41]. Up-to-date the majority of the Cypriot MEN2 patients and their relatives identified with various types of severe *RET* pathogenic variants underwent total thyroidectomy with central node dissection with the exception of a few that had a delayed clinical diagnosis and unfortunately passed away [11]. Information regarding the epidemiological frequencies of the identified *RET* pathogenic variants and their associated pathogenic effects can be obtained from the database *Leiden Open Variation Database* (https://databases.lovd.nl/shared/variants/RET) and the *Revised American Thyroid Association guidelines for the management of medullary thyroid carcinoma* [41].

#### Molecular diagnosis and research of premature and delayed puberty

Since 2016, *MGFT* at the *CING* offers specialized tests for the diagnosis of premature and delayed puberty. These tests are currently well established and up-to-date more than 400 have been performed using various genetic platforms that include NGS, Sanger sequencing and MLPA for the identification of possible genetic origin malformations that contribute to premature and delayed puberty.

##### *Premature puberty*

**Fig. 5:**
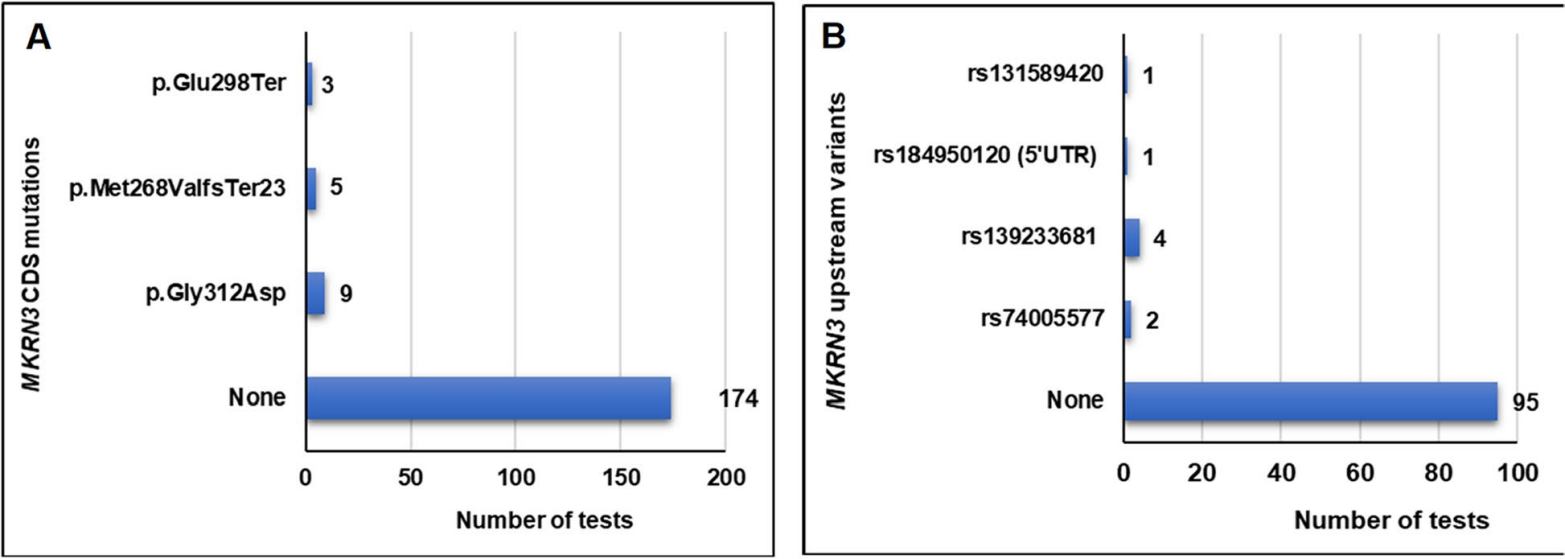
One hundred and ninety-one (191) children with clinical phenotype of CPP were tested for *MKRN3* pathogenic variants. **A** A total of three pathogenic variants in the *MKRN3* coding region have been found, with the novel p.Gly312Asp being the most frequent and thus suggesting a founder effect. **B** Four other rare upstream *MKRN3* likely pathogenic variants have also been found in 103 of the 191 children with CPP who have been tested

A total number of 191 of the above-mentioned tests examined the intronless *MKRN3* gene, where paternally inherited loss-of-function pathogenic variants are the leading cause of central precocious puberty (CPP) and that is mostly observed in girls (Fig. 5). Up-to date, in our examined cohort of clinically diagnosed female patients with CPP we identified specific novel and previously reported loss-of-function pathogenic variants in the coding region, the promoter and the 5’-UTR of the maternal imprinted *MKRN3* gene [4, 14, 16, 17]. Interestingly, nine girls from seven nonrelated families of Cypriot descent all shared the same novel loss-of-function p.Gly312Asp pathogenic variant, indicating another possible founder effect phenomenon [14, 17] (Fig. 5A). In 103 of the 191 tests, eight other CPP girls harbored likely pathogenic upstream variants in the *MKRN3* gene. More specifically, four of these girls with CPP had in the promoter upstream to the transcription start site the *rs139233681*, two the *rs74005577*, one the *rs131589420* and one the 5’UTR *rs18495012*0 [4, 17] (Fig. 5B). Whole exome sequencing (WES) was also performed in a selected group of forty-four CPP girls that were initially tested negative for *MKRN3* mutations. The results of this investigation were recently reported, which identified nine rare *DLK1* variants in eleven girls, two rare *KISS1* variants in six girls and two rare *MAGEL2* variants in five girls with CPP [17].

The pubertal process is in addition to imprinting and other mechanisms also controlled by epigenetic mechanisms such as DNA methylation at CpG dinucleotides in puberty-related genes. In a recent research study by *MGFT* at *CING* the methylation status of the *MKRN3* promoter in the hypothalamus of female mouse before, during and after puberty was investigated and we noted that CpG dinucleotide methylation was significantly lower within the CpG islet region at the pre-pubertal stage when compared with the pubertal and post-pubertal stage [5]. Moreover, in this same study, we also recognized by in silico analysis of the transcription factor binding sites on the *MKRN3* CpG islet the recruitment of 29 transcriptional regulators, 14 of which were transcriptional repressors [5]. All girls with CPP and that were genetically tested at the premises of *MGFT* and identified to carry causative *MKRN3* variants in the coding, promoter and the 5’-UTR region of the imprinted gene were initially confirmed with GnRH stimulated Luteinizing Hormone (LH) levels, and treatment was administered to them with long-acting GnRH analogue with effective regression of pubertal signs [4, 14, 16]. Our recent work in female mice of the methylation status of hypothalamic Mkrn3 promoter across puberty demonstrated that differentially methylated CpG dinucleotides located in the Mkrn3 promoter could influence the transcriptional activity in pre-pubertal stage by significantly lower its methylation levels. At present, in vitro studies by *MGFT* are underway aiming to delineate the possible mechanisms and the consequences of differential methylation of the Mkrn3 promoter.

##### *Delayed puberty*

In a recent report authored by the *MGFT* members and collaborators, a total of eight unrelated cases with congenital hypogonadotropic hypogonadism (CHH) and biochemically tested as GnRH deficient have been presented. All nine cases (six males and two females) underwent WES by NGS and were all found to carry seven novel and two previously reported pathogenic or likely pathogenic variants in a series of genes known to be implicated with the CHH/Kallmann syndrome and delayed puberty (Table 2) [12, 48–50]. The identified novel variants were initially examined by in silico tools and structural analysis of the predicted pathogenicity at the protein level was confirmed [12].

**Table 2:** Clinical and genetic characteristics of the tested patients with CHH with seven of them harboring novel mutations

Patient ID	Sex	Phenotype	Gene	Genotype	Inheritance	MAF (%) – gnomAD v2.1.1	Variant Classification	Previously described
1	M	CHH	*ANOS1*	p.Gln82*	X-linked	Absent	Pathogenic	-
2	M	KS	*WDR11*	p.Leu244Pro/WT	AD	Absent	Probably Pathogenic	-
3	M	CHH	*SRA1*	p.Ile179Thr/WT	AR	0.00081	Probably Pathogenic	[49, 52]
			*RNF216*	p.Asp792Asn/WT	AR	Absent	Probably Pathogenic	-
4	M	CHH	*CHD7*	p.Arg2400Trp/WT	AD	0.0000154	Probably Pathogenic	-
5	M	CHH	*FGFR1*	p.Pro186Ala/WT	AD	Absent	Probably Pathogenic	-
			*POLR3A*	p.Arg561Gly/WT	AR	Absent	Probably Pathogenic	-
6	M	CHH	*FGFR1*	p.Arg822Cys/WT	AD	0.00026	Probably Pathogenic	[50]
7	F	CHH	*SRA1*	p.Ile179Thr/p.Ile179Thr	AR	0.00081	Probably Pathogenic	[49, 52]
8	F	Delayed Puberty	*IGSF10*	p.Val1420Met	AD	0.000035	Probably Pathogenic	

#### Molecular diagnosis of disorders of sexual differentiation

For more than ten years, *MGFT* department is also offering DNA testing either by Sanger sequencing and WES with NGS for children born with incomplete genital or sexual development and for adults with genital abnormalities. Such cases include among others five unrelated Cypriot patients with 46,XY karyotype born with ambiguous genitalia and that were raised as females. When these patients underwent genetic screening of the *SRD5A2* gene proved to carry particular pathogenic variants known to the be the cause of 5α reductase deficiency [6, 51]. In the report by *MGFT* that followed the above genetic diagnosis verified the clinical diagnosis of 5α reductase deficiency, we also postulated that the IVS1-2A > G pathogenic variant may correspond to a founder effect phenomenon as a result of its high carrier frequency (0.98%) that we observed in the Cypriot population [51]. The clinical, biochemical and genetic features of two nonrelated newborns with 17β-HSD-3 deficiency of Cypriot and Greek origin were also presented by our department back in 2012 and in 2018. Genetic diagnosis for both cases was confirmed after had been found to carry in compound heterozygosity novel and previously reported pathogenic variants in the *HSD17B3* gene. Immediately, after the genetic diagnosis they both underwent through successful surgical correction of cryptorchidism and hypospadias [7, 8]. Recently, using WES with NGS we were able to reveal in a 46,XY Greek-Roma boy from a consanguineous family a homozygous pathogenic variant in the *HSD3B2* gene that is known to be causing 3β-Hydroxysteroid dehydrogenase (3β-HSD) deficiency [10]. In a similar fashion where consanguinity is an issue another phenotypically female with a 46,XY karyotype patient of Syrian ethnic background was identified with the severe and known p.Arg164Pro pathogenic variant in the *DHH* gene [9]. All of the above DSD patients including their families following the genetic diagnosis went through appropriate genetic counselling by our genetic counselors and a few through surgical treatment where possible.

#### Molecular diagnosis of glucose and insulin homeostasis – MODY and obesity

The department of *MGFT* at CING currently offers molecular investigation of glucose and insulin homeostasis defects that includes among others MODY, melanocortin-4 receptor (MC4R) deficiency and obesity by using an in silico panel from WES of 58 genes (*ABCC8 ADCY3 AGPAT2 ALMS1 BLK BSCL2 CEL CISD2 DCAF17 DMXL2 EIF2AK3 FOXC2 FOXP3 FTO GATA4 GATA6 GCK GLIS3 HAMP HFE HJV HNF1A HNF1B HNF4A IER3IP1 IL2RA INS INSR KCNJ11 KLF11 LMNA LRBA MC4R MNX1 MT-TL1 NEUROD1 NEUROG3 NKX2-2 PAX4 PCBD1 PDX1 PIK3R1 PLIN1 POLD1 PPARG PTF1A RFX6 SLC19A2 SLC29A3 SLC2A2 SLC40A1 STAT1 STAT3 TFR2 TRMT10A WFS1 ZBTB20 ZFP57*) upon request for an exhaustive investigation. Right up to the present time, more than > 30 unrelated male and female patients of various ages ranging from neonatal to adult age with clinical and biochemical criteria of glucose and insulin homeostasis have been genetically screened for mutations using the above mentioned in silico panel from WES of 58 genes. All of these patients were recruited from the Archbishop Makarios III Hospital (Nicosia, Cyprus), the Paedi Center for Specialized Paediatrics (Nicosia, Cyprus) and the Aretaeio Hospital (Nicosia, Cyprus). Currently, using the above in silico panel from WES of 58 genes, a total of 4 patients with MODY 2 and 2 patients with MODY 3 were respectively identified to carry known pathogenic variants in the *GCK* and the *HNF1A* genes (unpublished data). The age of these patients identified with the pathogenic variants in the *GCK* and *HNF1A* genes ranged between 10–28 years at time of diagnosis with an average borderline fasting blood sugar of 113 mg/dl. The Oral Glucose Tolerance Test (OGTT) regarding the above patients with 1.75 g/kg showed an increase of an average blood glucose from 123 mg/dl to 154 mg/dl at 2 h with also an average of corresponding insulin levels of 13.5 μlU/mL and 36.5 μlU/mL, respectively (unpublished data). In a recent study by our department that investigated the implication of *ADCY3* gene variants as candidate for the regulation of body weight, 33 severely obese adolescents and young adults (18 females and 15 males aged 15–20 yrs) were included in the study [19]. All of these patients had BMI >  + 2.5 standard deviation score (SDS) at the time of genetic testing and were diagnosed with early‑onset obesity defined as BMI >  + 2 SDS from the age of 3 years onwards. According to their medical records, patients were not diagnosed with any other underlying medical conditions. A total of 51 age‑matched non‑obese individuals (40 females and 11 males) of Greek‑Cypriot origin were also included as a control group. The genetic screening of *ADCY3* revealed a total of five heterozygous recessive variants in patients, four of which were previously reported. A novel variant that involves a heterozygous c.349 T > A change in exon 1 of the gene locus, leading to a missense p.Leu117Met substitution was found in two unrelated patients. As a result of the fact that monogenic obesity is extremely rare, our findings in the above targeted patients diagnosed with severe obesity since childhood provided strong indication that *ADCY3* is a significant mediator of energy homeostasis with a conceivable role in the growth of obesity.

## Conclusions

The present report aimed to recapitulate the collected data of inherited endocrine diseases of the last fifteen years from an ENDO-ERN Reference Center such as the *MGFT* department at the *CING*. The data presented here emerged is the consequence of sophisticated diagnostic approaches and research activities and highlight the main objective of the European Reference Networks, which is to ensure accessible diagnosis and eventually develop rational treatment strategies and cohesive management plans.

## Data Availability

The authors confirm that all presented data of the current study is a summary of previous cumulative work of our group and data sharing is not applicable.

## Abbreviations

*MGFT*: Molecular Genetics-Function and Therapy
*CING*: Cyprus Institute of Neurology and Genetics
Endo-ERN: European Reference Network on Rare Endocrine Conditions
CAH: Congenital Adrenal Hyperplasia
MEN: Multiple Endocrine Neoplasia
MEN2: Multiple endocrine neoplasia type 2
DSD: Disorders of Sexual Differentiation
NGS: Next-generation sequencing
MODY: Maturity onset diabetes of the young
21-OHD: 21-Hydroxylase deficiency
SW: Severe salt-wasting
SV: Simple virilizing
17-OHP: 17-OH Progesterone
MLPA: Multiplex ligation-dependent probe amplification
EuRRECa: European Registries for Rare Endocrine Conditions
Pdex: Prenatal dexamethasone
EMQN: European Molecular Genetics Quality Network
FMTC: Familial medullary thyroid carcinoma
ATA: American Thyroid Association
ETA: European Thyroid Association
MTC: Medullary thyroid carcinoma
AD: Autosomal dominant
CPP: Central precocious puberty
WES: Whole exome sequencing
CHH: Congenital hypogonadotropic hypogonadism
MC4R: Melanocortin-4 receptor
OGTT: Oral Glucose Tolerance Test
SDS: Standard deviation score
